# How Protons Move in Enzymes—The Case of Nitrogenase

**DOI:** 10.1021/acs.jpcb.2c08567

**Published:** 2023-03-02

**Authors:** Per E. M. Siegbahn

**Affiliations:** Department of Organic Chemistry, Arrhenius Laboratory, Stockholm University, SE-106 91 Stockholm, Sweden

## Abstract

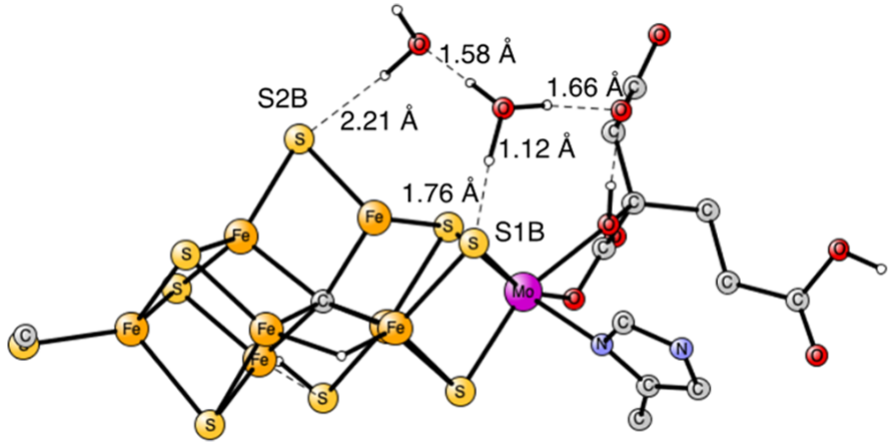

When moving protons in enzymes, water molecules are often used as intermediates. The water molecules used are not necessarily seen in the crystal structures if they move around at high rates. In a different situation, for metal containing cofactors in enzymes, it is sometimes necessary to move protons on the cofactor from the position they enter the cofactor to another position where the energy is lower. That is, for example, the situation in nitrogenase. In recent studies on that enzyme, prohibitively high barriers were sometimes found for transferring protons, and that was used as a strong argument against mechanisms where a sulfide is lost in the mechanism. A high barrier could be due to nonoptimal distances and angles at the transition state. In the present study, possibilities are investigated to use water molecules to reduce these barriers. The study is very general and could have been done for many other enzymes. The effect of water was found to be very large in the case of nitrogenase with a lowering of one barrier from 15.6 kcal/mol down to essentially zero. It is concluded that the effect of water molecules must be taken into account for meaningful results.

## I Introduction

In redox enzymes, electrons commonly have to be transferred over long distances. That occurs in steps with each step being up to about 10 Å, following well-known rules.^1^ In general, protons also have to move over long distances. Analysis of X-ray structures shows that there are sometimes well-defined proton transfer chains with typically carboxylates and imidazoles placed at appropriate distances from each other allowing fast proton transfer. However, well-defined proton transfer chains are not always present. Cytochrome c oxidase is an example where protons have to move long distances, over 10 Å, with no apparent proton acceptors.^2^ In that case it is known that water molecules are used for transfer of the protons. Those water molecules are not seen in the X-ray structures since they move around fast between different positions.

**Figure 1:**
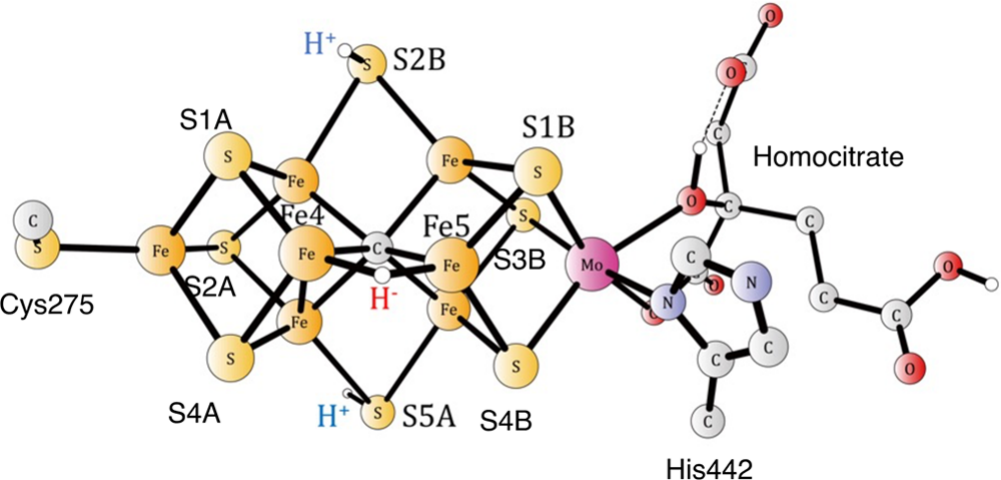
Previously optimized E_1_ state, where the S3A sulfide was found to be lost.^12^ Some hydrogens and atoms outside the cofactor are not shown.

Nitrogenase is the only enzyme in nature able to fix nitrogen from the air and turn it into biologically useful products. It is a typical redox enzyme but with an unusual cofactor where nitrogen becomes bound.^3^ The cofactor FeMoco is shown in Figure 1.

Since the present paper is intended to be a rather general paper, the details of the nitrogenase mechanism will not be described here. In short, the catalytic cycle starts with four reduction steps, E_0_ to E_4_, with an addition of a (H^+^, e^–^)-couple in each reduction. In E_4_, N_2_ is activated and one H_2_ is released.^4,5^ After the N_2_ activation, there are six more reduction steps in which N_2_ is protonated to form two NH_3_ molecules.

The mechanism for electron transfer to the cofactor is complicated involving two ATP for each electron and the binding between two different proteins.^5^

For the proton transfer to the cofactor, which is the topic of the present paper, one long known pathway ends at His195 which is hydrogen bonded to S2B of the cofactor. However, Dance suggested that His195 in that pathway is used only once since he found that the barrier is too high for rotating the histidine for the further proton transfers to the cofactor.^6,7^ Instead, he argued that the protons should be delivered to S3B of the cofactor. From S3B, the protons should be distributed to other positions of the cofactor via the other sulfides. Ryde et al. recently suggested that also S5A and S4B could be reached using the water molecule HOH-519, found in the crystal structure.^8^ Dance only considered proton transfers for the first four steps (E_0_ to E_4_) of the catalytic cycle, before N_2_ has become bound to the cofactor. Ryde et al. continued the study by looking also at the protonations of N_2_ in the steps E_5_ to E_8_.

During the past five years, experimental studies have indicated that a sulfide might be lost during catalysis.^9−11^ Furthermore, using model calculations, a mechanism for N_2_ activation was recently suggested in which it was found that a sulfide indeed could be lost with a low barrier, which occurred after four reductions of the cofactor prior to catalysis.^12^ It was found to be kinetically preferred compared to other pathways. In relation to these suggestions, Ryde et al. considered the proton transfer steps after the loss of a sulfide. They found that transferring protons to the bound substrate from S3B, S5A, and S4B had prohibitively high barriers if a sulfide was lost. Therefore, the conclusion they drew was that their calculations provide strong arguments against a mechanism for N_2_ reduction including a dissociation of a sulfide.

**Figure 2:**
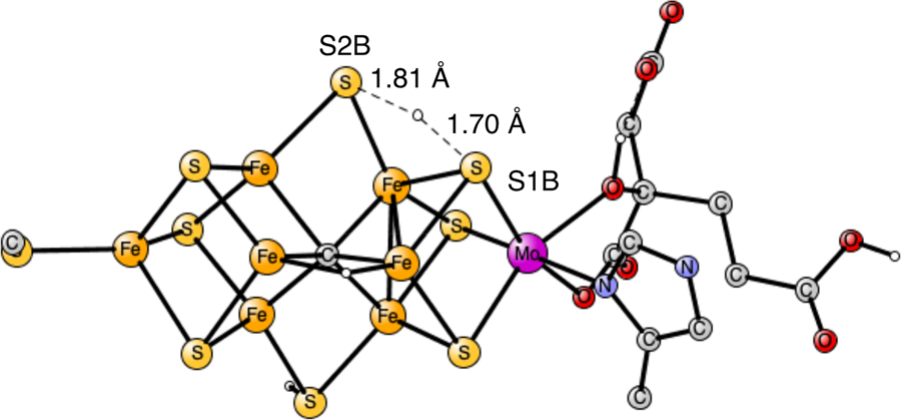
Transition state for proton transfer between S1B and S2B. The barrier is 15.6 kcal/mol.

Since water has been found to be a part of many mechanisms for proton transfer in enzymes, the present study was made to investigate if water could be used for moving protons on the cofactor for nitrogenase. The case of highest interest is where a sulfide has been lost. The state investigated for the present study was arbitrarily selected to be the E_1_ state previously optimized; see Figure 2.^12^ Ideally, a state where N_2_ is bound on the cluster should have been studied, but the present investigations of the mechanism have not yet reached that level for the case where a sulfide has been lost. Also, the present mechanism for N_2_ activation differs strongly at an early stage (E_1_ toE_4_) from the one of Ryde et al., why a direct comparison is anyway difficult to make. The general question asked here is if water molecules could affect the barriers for proton transfers between the different sites on the cofactor.

## II Methods

The methods used here are essentially the same as the ones used in earlier studies.^13−15^ The underlying method used is density functional theory with the B3LYP functional.^16^ In the original version of the B3LYP functional, a 20% fraction of exact exchange has been used. In applications on redox enzymes containing transition metals, it has been found that 15% exact exchange give significant improvements, and it has been used in the present study. The method has been bench-marked on several redox enzymes where it was found that the accuracy is in general about 3 kcal/mol.^15^ The bench-mark test included full redox mechanisms for Photosystem II, Cytochrome c Oxidase, NiFe and FeFe hydrogenases, NiFe-CO dehydrogenase, Multicopper oxidases, and Acetyl-CoA synthase. It is particularly noteworthy that the redox steps are very well reproduced.

The geometry optimizations and Hessian evaluations used the B3LYP functional with 20% exact exchange and a lacvp* basis set. For the final energies 15% exact exchange was used with a cc-pvtz(-f) basis for the first-row atoms, and a lacv3p* basis for the metals was used. Solvent effects were computed with a dielectric constant of 4.0, and dispersion effects were included using the D2 method.^17^ The Jaguar^18^ and Gaussian^19^ programs were used.

The description of the active site used the cluster method,^20^ and the model is the same as used in the most recent study of nitrogenase.^12^ It includes, besides the FeMoco with ligands, also His195, Arg96, Arg359, Glu380, Phe381, and Gln191. Since the positive region outside the homocitrate was left out, an additional proton was added on that ligand. The model contains about 170 atoms. The charge of the model is −2, and the spin-state is a triplet.

## III Results

In the present study, the effect of water molecules on barrier heights for proton transfer is studied. Nitrogenase is chosen as an example, but it could have been any enzyme, where protons are moved on the cofactors. The study is general and is not taken from a suggested specific part of the mechanism for nitrogenase. The previously optimized E_1_ state, where the S3A sulfide was found to be removed from the cofactor,^12^ is chosen as model, since proton transfers were recently studied for such a case. The best protonations found were on S2B and S5A. A hydride is present, bridging two of the irons.

In the first of the two examples given here, a proton has been delivered from the medium to S1B. Since the best protonation site is S2B, the proton needs to move to that position. The transition state for the straightforward proton transfer is shown in Figure 2. A rather large barrier of 15.6 kcal/mol was obtained in line with results obtained in the previous studies.^6−8^

To study the effect of water on the barrier, it is important to account for the cost of taking a water molecule from bulk water. An empirical value of 14 kcal/mol is used here as that cost.^13−15^ That value can be approximately derived from the experimental free energy for the binding of one water molecule to bulk water of −6.3 kcal/mol.^21^ A large entropy effect is obtained in that case since the water is moved from the gas phase. In the present case, essentially no such entropy effect should be present.

**Figure 3:**
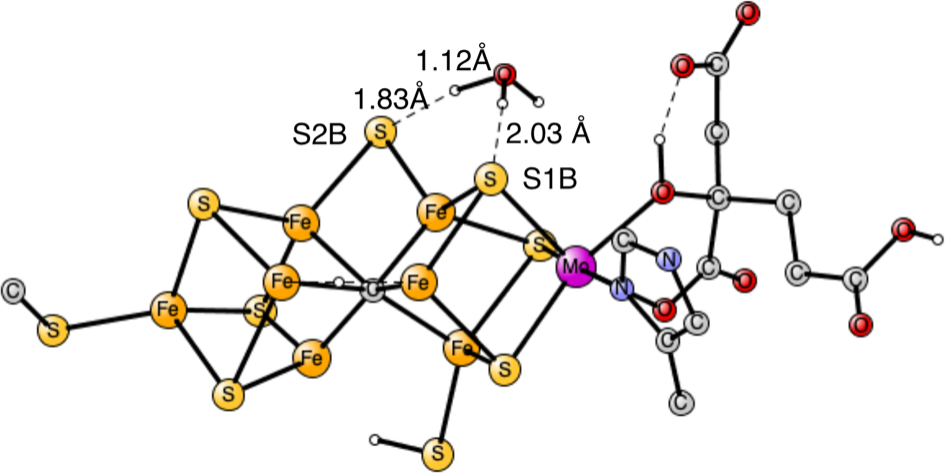
Transition state for proton transfer between S1B and S2B with the help of one water molecule. The barrier is 14.3 kcal/mol.

One water was first added in the region of S1B and S2B. The TS is shown in Figure 3. As seen in the figure, a H_3_O^+^ is formed. The hydrogen bond distances are 1.83 Å to S2B and 2.03 Å to S1B. The O–H bond distances are elongated with one of them being 1.12 Å. However, the lowering of the barrier by that addition is only 1.3 kcal/mol, from 15.6 to 14.3 kcal/mol. The cost of taking the water molecule from the medium is 2.3 kcal/mol and is included in the barrier height.

**Figure 4:**
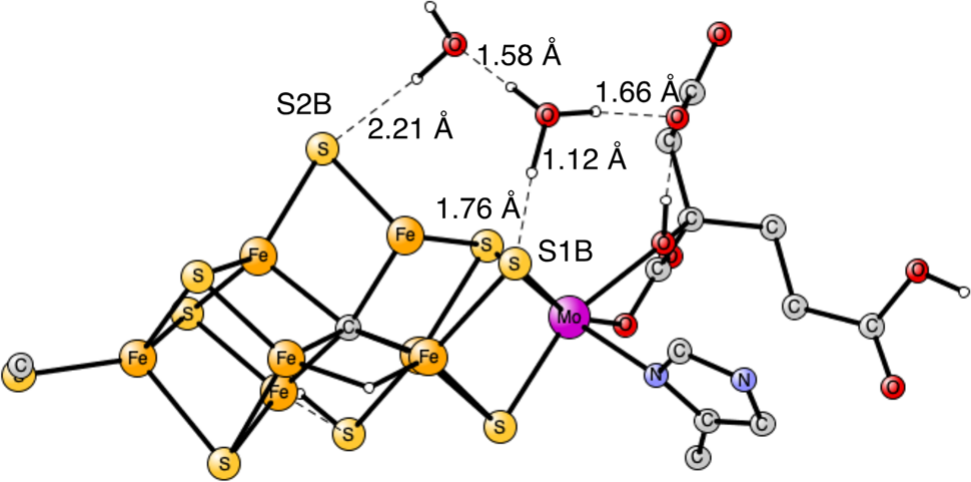
Transition state for proton transfer between S1B and S2B with the help of two water molecules. The barrier is 0 kcal/mol.

It is known that proton transfer chains can consist of several water molecules.^22^ Therefore, another water molecule was added. The TS is shown in Figure 4. Again, a H_3_O^+^ is formed at the TS. There is a strong hydrogen bond between the two waters with a distance of 1.58 Å. One of the O–H distances has increased to 1.12 Å. The hydrogen bond to S1B is quite strong with a distance of only 1.76 Å. A normal S–H bond distance is 1.4 Å. There is also a strong hydrogen bond to the homocitrate ligand with a distance of 1.66 Å. The effect of the second water is very large, and the barrier is now essentially zero. Therefore, the effect of considering water in the TS is as large, −15.6 kcal/mol compared to the situation in Figure 2 without any water. The binding of the second water is exergonic by −4.6 kcal/mol, which means that the two water molecules can be obtained from the medium without cost, since the cost of adding the first water is +2.3 kcal/mol.

**Figure 5:**
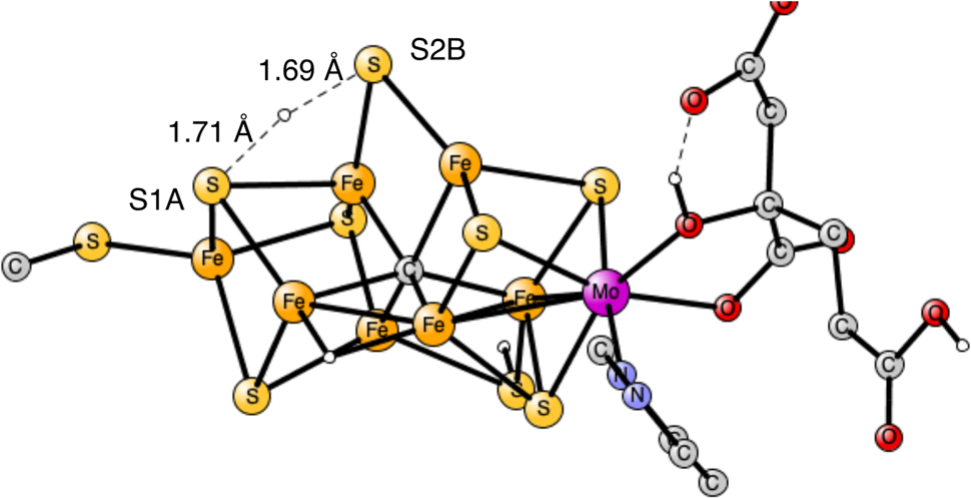
Transition state for proton transfer between S1A and S2B. The barrier is 9.5 kcal/mol.

The final part of the present investigation is another proton transfer from S1A to S2B. Again, a TS was obtained without any water molecule, see Figure 5, and was found to have a barrier of 9.5 kcal/mol. The proton is in between the two sulfides at the TS with distances of 1.71 and 1.69 Å.

**Figure 6:**
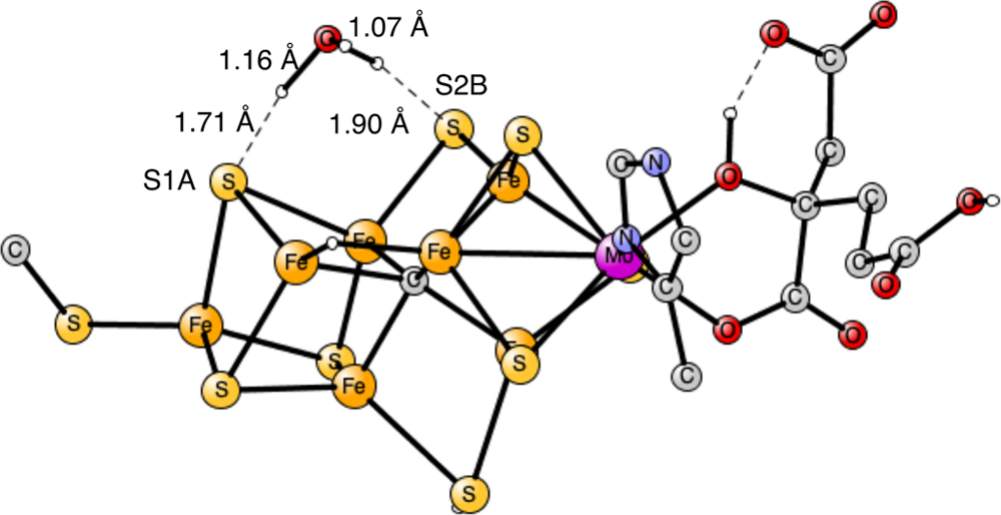
Transition state for proton transfer between S1A and S2B with the help of one water. The barrier is 4.0 kcal/mol.

When one water was added, the TS in Figure 6 was found. Again, a H_3_O^+^ was formed with one long O–H distance of 1.16 Å. The hydrogen bond to S1A is quite short with a distance of 1.71 Å. A cost of 0.7 kcal/mol was found to take the water from the medium, which is included in the barrier of 4.0 kcal/mol. The lowering effect on the barrier of 5.5 kcal/mol is not very large but still significant. In this case, adding another water did not lead to any lowering of the barrier as it did for the proton transfer between S1B and S2B.

## IV Conclusions

Two examples are discussed of the effect of adding water for proton transfer barriers. The main motivation for the present study is that water was not included in the proton transfer barriers in some recent studies of nitrogenase.^6−8^ Far reaching consequences were drawn for the mechanism of nitrogenase. For example, it was concluded that calculations provided strong arguments against a mechanism for N_2_ reduction including a dissociation of a sulfide.

The examples given here show very large effects on the barrier heights by including water in the mechanism for transferring the protons. In one of the two cases studied, the effect of adding two water molecules lowered the transfer barrier from 15.6 kcal/mol down to essentially zero. A general feature of the transition states is that H_3_O^+^ is formed. It should be emphasized that H_3_O^+^ is not present for the equilibrium structures. One reason the effect is so large is that more optimal angles for a TS can be obtained. It is also very likely that the effect will be even larger when the distance between donor and acceptor is large, such as in the case when protons have to be moved from the sulfides of the cofactor to the N_2_ substrate. In that case, proton transfers could even have a lower transfer barrier by moving them directly from the medium to the substrate.

The finding of a large effect of adding water for proton transfer barriers is not new and not limited to enzymes but is quite general when water is present as a medium. For example, in a study 25 years ago on the Wacker process, the importance of chains of water molecules was also found.^22^ Likewise, it was found to be important for proton transfer in photosystem II.^23^ It is important to point out that a presence of crystal water molecules in the X-ray structure is not necessary for water to be important in the transition state. In cytochrome c oxidase, there is a large area absent of crystal waters, through which it is known that protons are transported using water.^2^

## Supporting Information Available

Coordinates for all structures (PDF)
