# Supplementary material for: How Protons Move in Enzymes—The Case of Nitrogenase
Source: J Phys Chem B. 2023 Mar 2;127(10):2156–9. doi: 10.1021/acs.jpcb.2c08567 (PMC10026063; doi:10.1021/acs.jpcb.2c08567)
Supplement: Supplementary file 1 — jp2c08567_si_001.pdf [file jp2c08567_si_001.pdf]

Supporting information:

How Protons Move in Enzymes – the Case of Nitrogenase

Per E. M. Siegbahn

Department of Organic Chemistry, Arrhenius Laboratory, Stockholm University, SE-106 91, Stockholm, Sweden

The structure with a proton on S2B.   # denotes a fixed atom

Mo1	15.9445520263	-6.0672768122	53.2237585106
Fe2	8.1395967783	-7.8505600926	53.9630531205
Fe3	10.3616904622	-6.0081830760	54.6053732072
Fe4	10.5542537015	-7.1571844752	52.2635966526
Fe5	11.0690876293	-8.4645675081	54.3916382161
Fe6	13.6828262892	-7.8709445914	53.9996401857
Fe7	13.1976986462	-5.4669157178	54.9289494876
Fe8	13.1657464668	-6.0677203839	52.1269199444
C9	17.6390526046	-2.4877418880	54.2280202590
H10	17.9578590457	-1.5514190175	53.7666298501
H11	16.5438139954	-2.4661882131	54.3002399365
C12	18.0475533945	-3.6902089112	53.3515823398
C13	19.5905465728	-3.7084672849	53.1397245250
H14	20.0578062910	-3.9095333688	54.1088025651
H15	19.9007940612	-2.7071181765	52.8246651375
C16	20.0273170730	-4.7484005383	52.1011186064
H17	19.7246871526	-4.4263162255	51.0960160073
H18	19.5055865620	-5.6868981804	52.2803004846
C19	17.3676974733	-3.7348796418	51.9548885304
O20	16.8038558592	-4.8742958678	51.6574753556
O21	17.4833430999	-2.7740407864	51.1963698658
O22	17.6326363244	-4.8842131063	54.0502035543
C23	12.0252941525	-6.7893108989	53.7006782899
S24	9.5008672955	-7.7815970303	55.9803753807
S25	15.1729170746	-6.8100820973	55.4919742704
S26	8.8122284926	-5.5529065274	52.8913289260
S27	11.4951002458	-4.5579099254	56.3681866866
S29	14.2418922343	-4.1926569953	53.1894232857
S30	9.7161700920	-9.3506844679	52.5736812241
S31	14.7919789719	-7.7792140338	51.9073560967
S32	11.7992433959	-6.4643551558	50.0862183609
H33	13.6351748602#	-2.1902104270#	49.0425561874#
C34	13.2979254360#	-2.0241165046#	50.0550216883#
H35	13.7703888554	-2.7605771429	50.7090422669
H36	13.5642353714	-1.0164692265	50.3891451446

N37	11.8304580989	-2.2115717016	50.0341855841
H38	11.3436157232	-1.7912724848	49.2549730569
C39	11.0319712201	-2.8640241778	50.9029240732
N40	11.4193956229	-3.2657251943	52.0987290914
H41	10.8161903503	-3.9313356115	52.5969754589
H42	12.3852363646	-3.1992203746	52.4340721521
N43	9.7266064850	-3.0589936223	50.5609111546
H44	9.5278231921	-3.1974697397	49.5779119976
H45	9.2033406957	-3.6909478374	51.1840261305
H46	7.9310783042	0.3892944158	59.4128146987
C47	8.3259678113	-1.3587962573	60.6881412329
H48	8.2301195576	-1.5635189675	61.7621345327
H49	7.5161802858	-1.9078343779	60.1902449353
C50	9.6527060818	-1.8918802747	60.2445112401
N51	10.7579454257	-1.8242002658	61.0742773470
C52	9.9923689405	-2.4912094602	59.0540379344
H53	9.4271590818	-2.7473444194	58.1707939041
C54	11.7412718725	-2.3783773619	60.3905635782
H55	12.7550650333	-2.5206909972	60.7387147710
N56	11.3321562476	-2.7934423915	59.1614342681
H57	18.9733926873	-4.4847484063	57.4807971048
H58	16.6976613677	-8.9062129260	50.7328370311
C59	18.3332589255	-7.4978569354	50.8219503967
H60	17.7565755434	-6.5730481957	50.7492458653
H61	19.2900988802	-7.3268487530	50.3126289406
C62	18.6137031627	-7.7824992137	52.2729601704
N63	17.6902988815	-7.5126902193	53.2780033982
C64	19.7470900561	-8.2905119097	52.8609028517
H65	20.7002673112	-8.5653620803	52.4382963906
C66	18.2538294657	-7.8400829274	54.4292547282
H67	17.8136727426	-7.7018708296	55.4043642329
N68	19.4961136266	-8.3277061621	54.2227414993
H69	20.1564730195	-8.4925425975	54.9696985765
H70	6.3928523317#	-9.4883342661#	56.5258904352#
C71	6.2082082181	-9.8946566857	55.5236501010
H72	7.0553014225	-10.5294772961	55.2474777225
H73	5.3047117735	-10.5142491419	55.5529562977
S74	5.9547162756	-8.5514237626	54.2700802350
H75	11.8680867615	-3.2742022587	58.4446521217
H76	17.7899451208	-4.6672124195	55.0413657575
C78	18.2020547275	-2.5677561776	55.6711856245
O79	18.2501340480	-3.7569669411	56.1852711948
O80	18.5358166439	-1.5164174391	56.2436289162
C81	21.4830655395	-5.1227387452	52.0077777064
O82	21.9162658705	-6.0964309158	51.4161028497
O83	22.3208179998#	-4.2658020734#	52.6398598038#
H84	15.7024659672#	-0.1996311878#	61.4912310089#
C85	14.8549924193#	-0.0829920456#	60.8320024915#
H86	13.9165015895#	-0.2867338793#	61.3260937710#
H87	14.8242929124	0.9913623280	60.5831239689

C88	15.0599866108#	-0.8751942154#	59.5441988380#
H89	14.3562301059	-0.5256927626	58.7783975192
H90	14.8334269777	-1.9343862476	59.7155165673
C91	16.4930510699	-0.7775825619	59.0016217725
H92	16.8645034336	0.2553998122	59.0811516999
H93	16.5338124302	-1.0194764879	57.9335811455
C94	17.4782247126	-1.6972755340	59.7370400523
O95	17.2248719457	-2.2008857044	60.8370381595
N96	18.6599882402	-1.8907782980	59.0922925316
H97	19.2173912423	-2.6585429409	59.4483877351
H98	18.7156864189	-1.7265919279	58.0765726628
C99	10.6519924866#	0.0100039600#	53.3460013868#
H100	11.1636988285#	0.9592721287#	53.2872695872#
H101	9.5863320195#	0.1850131508#	53.3579375374#
H102	10.9070245070	-0.5629885676	52.4495086751
C103	11.0712947560	-0.7229169570	54.6349918890
H104	10.6726313446	-1.7456886013	54.5933031892
C105	10.4579412591	-0.0398249632	55.8660050858
H106	9.3627738301	-0.0096907228	55.7990903491
H107	10.8172088085	0.9950424423	55.9589676343
H108	10.7195605396	-0.5696000280	56.7881120781
C109	12.5989172744	-0.8092913362	54.7653734565
H110	13.0616542985	-1.3199164448	53.9128575548
H111	12.8890100677	-1.3638022414	55.6649345159
H112	13.0384508889	0.1959903285	54.8333399691
H113	13.4550772768#	-12.2303889064#	53.1883132394#
C114	13.9110026351#	-11.6210550751#	52.4219877800#
H115	14.6185786393	-10.9208168011	52.8795027402
H116	14.4155735364	-12.2531467861	51.6869082325
N117	12.7903315665	-10.9179918758	51.7900378303
H118	11.9872436180	-10.6731464622	52.3791992274
C119	12.8265589961	-10.3091683876	50.6098497435
N120	13.9934452729	-10.2790481240	49.8992545328
H121	13.9461546630	-9.7209709544	49.0542205786
H122	14.7983442961	-10.0094118234	50.4616465114
N123	11.6980867212	-9.8254890994	50.0700796760
H124	10.8808959952	-9.7763043627	50.7099296912
H125	11.7884798607	-8.9722036157	49.5213527639
H126	19.1962349434	-8.2132316992	61.7060182499
C127	19.5978935935#	-7.2298797992#	61.9900499059#
H128	20.6399478397	-7.3722491223	62.2942184109
C129	18.8327958495	-6.6935201304	63.1990056033
O130	19.3834316269	-6.2363021997	64.1974426097
C131	19.4918719078#	-6.2717978935#	60.7990634884#
H132	19.9353283740	-5.3044403335	61.0596895700
H133	18.4371807780	-6.0660677685	60.5903514720
C134	20.1385794008	-6.8235517856	59.5133893638
H135	19.8924702637	-7.8844650202	59.3840819451
H136	21.2353355043	-6.7807842402	59.5852518622
C137	19.7451604685	-6.1485126176	58.1926950534

O138	19.8586635777	-6.7261886943	57.1199619145
O139	19.3020425876	-4.9064049499	58.3464748388
N140	17.4778823733	-6.7533366689	63.0387861406
H141	17.1253488179	-7.0453801969	62.1356615612
C142	16.5368851993#	-6.0058841355#	63.8530416259#
H143	15.8283424107	-6.7005087328	64.3265092852
H144	17.1146663213	-5.5285418269	64.6482462201
C145	15.7632551207	-4.9560779307	63.0189132957
H146	16.4425709331	-4.1477227580	62.7279430429
H147	14.9936323415	-4.5135125126	63.6663397664
C148	15.1266837017	-5.5422540483	61.7689628516
C149	14.0489021124	-6.4403605102	61.8454722142
H150	13.6288164109	-6.6955925980	62.8173057189
C151	15.6384248101	-5.2194214907	60.5010797521
H152	16.4517848286	-4.5018262466	60.4281208560
C153	13.4965297374	-6.9970703720	60.6889635773
H154	12.6544246457	-7.6803549457	60.7660621395
C155	15.0883692371	-5.7777961425	59.3414077242
H156	15.4894830943	-5.5204193448	58.3650721434
C157	14.0167895286	-6.6699877052	59.4321525367
H158	13.5937012215	-7.0984377821	58.5279793433
H159	9.0037565857#	0.7050725615#	60.7752691766#
C160	8.1269785509#	0.1560808112#	60.4650009826#
H161	7.2836719644#	0.4592430887#	61.0677694085#
H162	11.3337522251	-5.6225223060	57.1848245973
H164	12.6853045136	-9.3057181763	54.4603007251
H165	18.2592776563#	-9.4987532744#	50.1505404583#
C166	17.5839763691#	-8.6559201780#	50.1520242596#
H167	17.2773626787#	-8.4419891651#	49.1387987601#
H169	23.2151189598	-4.6299714527	52.4987608505
H170	10.9914751586	-5.3997734267	49.8908249567

The structure with a proton on S1B.

Mo1	16.1476006913	-6.1530150587	53.3272453312
Fe2	8.0922890485	-8.1902118909	53.9007355461
Fe3	10.1548471934	-6.1111757919	54.5940921503
Fe4	10.6431424802	-7.4614468088	52.3615538615
Fe5	11.0001128030	-8.5831131431	54.6467170046
Fe6	13.5936120708	-7.9725574781	54.2165854651
Fe7	12.8403927441	-5.5274134726	55.0208215226
Fe8	13.1917312942	-6.1609330362	52.2886381171
C9	17.6560254615	-2.4885259111	54.2265639368
H10	17.9179360235	-1.5359517697	53.7628784967
H11	16.5641758699	-2.5146865256	54.3360243567
C12	18.0856677295	-3.6689896176	53.3281497014

C13	19.6052771065	-3.5906477295	53.0091859250
H14	20.1520915927	-3.7102472997	53.9496514159
H15	19.8190890137	-2.5878518461	52.6251389269
C16	20.0362611815	-4.6534602203	51.9940595506
H17	19.6498089980	-4.4185302068	50.9953703545
H18	19.5825610918	-5.6086667906	52.2640100656
C19	17.3129489976	-3.7552700240	51.9836298092
O20	16.7908639235	-4.9278923857	51.7180960707
O21	17.3118989147	-2.7916228548	51.2228415583
O22	17.8067179822	-4.8866492355	54.0534010073
C23	11.9521437421	-6.9486047521	53.8041139904
S24	9.2818797780	-7.8802409342	56.0021396086
S25	15.4110951933	-7.0297837863	55.6768876793
S26	8.7239445983	-6.0230793239	52.6843983965
S27	11.1107041087	-4.2667632205	55.6794711017
S29	14.3832850653	-4.4378372221	53.5483234829
S30	9.8932781826	-9.6748314835	52.7638921659
S31	14.8684370226	-7.9223312716	52.2008832113
S32	11.8810187740	-6.5485024909	50.2327704283
H33	13.6351747607#	-2.1902104119#	49.0425561518#
C34	13.2979255247#	-2.0241165232#	50.0550217718#
H35	13.9557356083	-2.5911351437	50.7189575509
H36	13.3450257481	-0.9600060595	50.3075139883
N37	11.9089885796	-2.5293422981	50.1101808117
H38	11.4342006878	-2.5988822659	49.2227675955
C39	11.2223718640	-3.0213365303	51.1681270791
N40	11.7713978045	-3.1121499195	52.3692261366
H41	11.2566771664	-3.4924373559	53.1720915381
H42	12.7803794730	-3.0881594390	52.5112036274
N43	9.9238663237	-3.3646060501	51.0180834276
H44	9.5317240123	-3.3772981487	50.0866864757
H45	9.5215474176	-4.0840339406	51.6515624068
H46	7.9263626115	0.3835999372	59.4124013002
C47	8.2895938512	-1.3595842136	60.6990436587
H48	8.3642816124	-1.5316910429	61.7814836767
H49	7.3713735415	-1.8628772760	60.3671638708
C50	9.4671141635	-2.0043580840	60.0401017273
N51	10.7304056093	-1.9510672741	60.6004380653
C52	9.4984795659	-2.7340325061	58.8737106188
H53	8.7381219728	-3.0109400684	58.1588080607
C54	11.5002354981	-2.6427122570	59.7785568716
H55	12.5577593185	-2.8252981504	59.9117222093
N56	10.8033869817	-3.1369948410	58.7211940047
H57	19.0752344360	-4.4565499108	57.4827766895
H58	16.7093344271	-8.8842701346	50.7583220947
C59	18.3755575825	-7.4912303339	50.7591384032
H60	17.7859832507	-6.5717568072	50.7437979375
H61	19.2783857147	-7.3073286996	50.1625820276
C62	18.8004446504	-7.7738789032	52.1714377584
N63	17.9812788222	-7.5118498544	53.2638994602

C64	20.0005055326	-8.2518650886	52.6398100239
H65	20.9077323468	-8.5206116155	52.1222658000
C66	18.6747204064	-7.8038145773	54.3526853125
H67	18.3684703049	-7.6374101030	55.3735767155
N68	19.8982588162	-8.2699690557	54.0199383473
H69	20.6286823621	-8.4619862125	54.6902320097
H70	6.3928523520#	-9.4883342410#	56.5258904048#
C71	6.1515832361	-10.0897977253	55.6422094698
H72	6.9476881573	-10.8262668410	55.4979358194
H73	5.2102231841	-10.6224644671	55.8189445960
S74	5.9471275393	-9.0351422909	54.1330182411
H75	11.1461681127	-3.6683512715	57.9176661857
H76	17.9927638957	-4.6320085149	55.0460440581
C78	18.2605124105	-2.5473781301	55.6522480326
O79	18.3912952168	-3.7409395965	56.1490606104
O80	18.5422546787	-1.4891165732	56.2328548505
C81	21.4994048183	-4.9681077002	51.8235441611
O82	21.9437786393	-5.7966885312	51.0485195131
O83	22.3208179626#	-4.2658020708#	52.6398597994#
H84	15.7024659237#	-0.1996312671#	61.4912310507#
C85	14.8549925411#	-0.0829919330#	60.8320024416#
H86	13.9165015895#	-0.2867338793#	61.3260937710#
H87	14.8267657079	0.9912473600	60.5823266760
C88	15.0599865306#	-0.8751942261#	59.5441988517#
H89	14.3614251704	-0.5211295509	58.7759734121
H90	14.8253146017	-1.9325997955	59.7130893046
C91	16.4945274948	-0.7870925181	59.0066217258
H92	16.8772319916	0.2408468273	59.0983406405
H93	16.5343176405	-1.0167155862	57.9354786764
C94	17.4695867109	-1.7249351049	59.7309334301
O95	17.2076515932	-2.2575694364	60.8141374217
N96	18.6603682265	-1.9012464221	59.0945315643
H97	19.2139030005	-2.6773710575	59.4379287885
H98	18.7241464362	-1.7153994388	58.0852151236
C99	10.6519925266#	0.0100038886#	53.3460012894#
H100	11.1636988171#	0.9592721365#	53.2872696142#
H101	9.5863320240#	0.1850131750#	53.3579375802#
H102	10.9182380586	-0.5865382668	52.4697218275
C103	11.0750036161	-0.6551363535	54.6685322963
H104	10.7285535886	-1.6943185942	54.6783256140
C105	10.4173758772	0.0518643717	55.8620216248
H106	9.3229702543	0.0256471966	55.7844870063
H107	10.7250087303	1.1065355387	55.9178149096
H108	10.6919415642	-0.4313768643	56.8056557421
C109	12.6011574123	-0.6649966865	54.8252252455
H110	13.0983131059	-1.1374096364	53.9704422293
H111	12.9004254943	-1.2198052852	55.7203651639
H112	12.9900315708	0.3603736944	54.9104592682
H113	13.4550772559#	-12.2303888923#	53.1883132381#
C114	13.9110026848#	-11.6210550780#	52.4219877722#

H115	14.5914492354	-10.8958099591	52.8799708866
H116	14.4485467222	-12.2496008125	51.7063314267
N117	12.7831843193	-10.9584458468	51.7612105051
H118	11.9324499429	-10.7735833370	52.3197179948
C119	12.8143947398	-10.3488325844	50.5844829720
N120	13.9984999851	-10.2011660253	49.9227498466
H121	13.9234803041	-9.6687287635	49.0631886996
H122	14.7406813816	-9.8323701482	50.5199505940
N123	11.6620680784	-9.9537653230	50.0029572897
H124	10.8429007583	-9.9876268328	50.6324643832
H125	11.7046176684	-9.0539917755	49.5234959441
H126	19.2024485305	-8.2159409539	61.7064666596
C127	19.5978935786#	-7.2298797842#	61.9900499108#
H128	20.6395813022	-7.3655061202	62.2983233797
C129	18.8295580393	-6.6917653777	63.1955257047
O130	19.3801436168	-6.2320912804	64.1928467550
C131	19.4918719028#	-6.2717978959#	60.7990634724#
H132	19.8979777689	-5.2918945308	61.0729795676
H133	18.4366562120	-6.0968749182	60.5640868189
C134	20.1915997220	-6.7981127543	59.5309239490
H135	19.9842941618	-7.8653792994	59.3888087687
H136	21.2836603472	-6.7176230071	59.6353924616
C137	19.8136703797	-6.1251733899	58.2071519316
O138	19.9243058815	-6.7079061183	57.1361225598
O139	19.3859087548	-4.8774327507	58.3533304043
N140	17.4754658647	-6.7513882799	63.0346875871
H141	17.1192112850	-7.0523145767	62.1359448306
C142	16.5368852138#	-6.0058841460#	63.8530416234#
H143	15.8469783614	-6.7041911783	64.3482499792
H144	17.1193011519	-5.5069763190	64.6315343387
C145	15.7309101880	-4.9853071125	63.0178645516
H146	16.3907307146	-4.1748389526	62.6899747323
H147	14.9716187023	-4.5388560582	63.6745087105
C148	15.0710499508	-5.6142261227	61.8031891874
C149	14.0035266105	-6.5176075154	61.9364048065
H150	13.6117995442	-6.7440486392	62.9269160807
C151	15.5451014383	-5.3248473955	60.5134646248
H152	16.3451033358	-4.5979630742	60.3953645369
C153	13.4214718586	-7.1105664795	60.8141109449
H154	12.5828917415	-7.7916416855	60.9338699053
C155	14.9663191832	-5.9217946087	59.3876971590
H156	15.3388391721	-5.6860006498	58.3953189660
C157	13.9030000225	-6.8163257729	59.5341879474
H158	13.4443720039	-7.2662822179	58.6581970370
H159	9.0037565692#	0.7050725935#	60.7752691667#
C160	8.1269785732#	0.1560807832#	60.4650009730#
H161	7.2836719645#	0.4592430664#	61.0677694199#
H162	15.8827018786	-8.2976288340	55.7650314666
H164	12.6780107455	-9.3554232386	54.8958666178
H165	18.2592776501#	-9.4987532794#	50.1505404456#

C166	17.5839763222#	-8.6559201725#	50.1520243187#
H167	17.2773626991#	-8.4419891678#	49.1387987534#
H169	23.2200174977	-4.5869497814	52.4383606292
H170	10.8699102145	-5.6559616719	50.2192563577

The TS structure with one water and a proton on S1B.

Mo1	16.0222750000	-5.9809110000	53.2622750000
Fe2	8.1865770000	-8.0625310000	54.0779670000
Fe3	10.4069480000	-6.0557250000	54.7700250000
Fe4	10.5629420000	-7.4083110000	52.4945360000
Fe5	11.1683800000	-8.6271860000	54.6649800000
Fe6	13.7643770000	-8.0277930000	54.1094350000
Fe7	13.1261140000	-5.5982400000	54.5190690000
Fe8	13.1390970000	-6.1998040000	51.8916360000
C9	17.7739220000	-2.3765330000	54.2541990000
H10	18.1674530000	-1.4695530000	53.7922660000
H11	16.6806440000	-2.2706420000	54.2849280000
C12	18.1250370000	-3.6165630000	53.4087340000
C13	19.6607430000	-3.7130150000	53.2091280000
H14	20.1131640000	-3.9439610000	54.1790090000
H15	20.0285680000	-2.7305280000	52.8976230000
C16	20.0416590000	-4.7702960000	52.1690640000
H17	19.7504740000	-4.4319470000	51.1656420000
H18	19.4889860000	-5.6895380000	52.3464210000
C19	17.4707250000	-3.6697230000	52.0036410000
O20	16.9464980000	-4.8254660000	51.6990450000
O21	17.5852010000	-2.7091120000	51.2476940000
O22	17.6506030000	-4.7739180000	54.1281120000
C23	12.0603770000	-6.9590990000	53.6026960000
S24	9.5292260000	-7.8519070000	56.1183210000
S25	15.1862530000	-6.6599510000	55.5502650000
S26	8.8145090000	-5.8654840000	52.9323100000
S27	11.6586060000	-4.2784400000	55.7650590000
S28	14.3041540000	-4.2099230000	53.0345510000
S29	9.8643950000	-9.6182850000	52.8312110000
S30	14.8879380000	-7.7771610000	52.0144700000
S31	11.9447640000	-6.2628720000	49.7564060000
H32	13.6351750000	-2.1902110000	49.0425560000
C33	13.2979260000	-2.0241160000	50.0550220000
H34	13.9914010000	-2.5016920000	50.7534360000
H35	13.1995580000	-0.9568210000	50.2672790000
N36	11.9607990000	-2.6771950000	50.0216770000
H37	11.8410430000	-3.3085540000	49.2373300000
C38	11.1786250000	-3.0411080000	51.0669080000
N39	11.5777460000	-2.8778700000	52.3231640000

H40	10.9889170000	-3.2136470000	53.0829050000
H41	12.5769420000	-2.9594930000	52.5295200000
N42	9.9353880000	-3.4917330000	50.8246160000
H43	9.6526940000	-3.6083170000	49.8616230000
H44	9.5022750000	-4.1485010000	51.4999850000
H45	7.9253870000	0.3593250000	59.4081370000
C46	8.3059840000	-1.3502840000	60.7444800000
H47	8.4460660000	-1.4735870000	61.8278950000
H48	7.3740580000	-1.8732370000	60.4910810000
C49	9.4413010000	-2.0107960000	60.0333300000
N50	10.7576280000	-1.8015140000	60.3984890000
C51	9.3769160000	-2.8892520000	58.9767510000
H52	8.5489180000	-3.3043830000	58.4207150000
C53	11.4664900000	-2.5459390000	59.5679510000
H54	12.5440460000	-2.6389490000	59.5679020000
N55	10.6776260000	-3.2278760000	58.6944730000
H56	19.0370530000	-4.4245380000	57.5245140000
H57	16.7028780000	-8.9379980000	50.7259780000
C58	18.3227230000	-7.5044840000	50.8425630000
H59	17.7471620000	-6.5791120000	50.7834690000
H60	19.2777780000	-7.3226660000	50.3327930000
C61	18.6184300000	-7.7985140000	52.2899330000
N62	17.7614010000	-7.4325890000	53.3241250000
C63	19.7302700000	-8.3827470000	52.8483450000
H64	20.6451440000	-8.7360960000	52.3999210000
C65	18.3416400000	-7.7770130000	54.4615210000
H66	17.9532120000	-7.5899510000	55.4503540000
N67	19.5337210000	-8.3647150000	54.2180350000
H68	20.1978710000	-8.6029680000	54.9403750000
H69	6.3928520000	-9.4883340000	56.5258900000
C70	6.1300790000	-10.0052920000	55.5976990000
H71	6.8873030000	-10.7692430000	55.3955660000
H72	5.1609590000	-10.5003260000	55.7266170000
S73	6.0001780000	-8.8078780000	54.1925550000
H74	10.9704640000	-3.8084010000	57.9067320000
H75	17.8188800000	-4.5451170000	55.1215790000
C76	18.2911090000	-2.4701220000	55.7159990000
O77	18.2632960000	-3.6559710000	56.2441980000
O78	18.6579790000	-1.4355950000	56.2903740000
C79	21.4854560000	-5.1703800000	52.0803700000
O80	21.9104570000	-6.1807410000	51.5501740000
O81	22.3208180000	-4.2658020000	52.6398600000
H82	15.7024660000	-0.1996310000	61.4912310000
C83	14.8549930000	-0.0829920000	60.8320020000
H84	13.9165020000	-0.2867340000	61.3260940000
H85	14.8287390000	0.9920070000	60.5855270000
C86	15.0599860000	-0.8751940000	59.5441990000
H87	14.3701170000	-0.5095060000	58.7728720000
H88	14.8095460000	-1.9284510000	59.7161420000
C89	16.4963500000	-0.8155700000	59.0137750000

H90	16.9039710000	0.2027320000	59.0997620000
H91	16.5360880000	-1.0524660000	57.9437730000
C92	17.4464060000	-1.7760400000	59.7419440000
O93	17.1283710000	-2.3760350000	60.7713040000
N94	18.6805140000	-1.8861300000	59.1725120000
H95	19.2191340000	-2.6812590000	59.4962580000
H96	18.7808240000	-1.6685210000	58.1732150000
C97	10.6519930000	0.0100040000	53.3460010000
H98	11.1636990000	0.9592720000	53.2872700000
H99	9.5863320000	0.1850130000	53.3579380000
H100	10.9267720000	-0.5981250000	52.4853120000
C101	11.0694570000	-0.6237560000	54.6901280000
H102	10.7491130000	-1.6720430000	54.7219110000
C103	10.3815850000	0.0962410000	55.8605910000
H104	9.2891200000	0.0563520000	55.7637650000
H105	10.6750230000	1.1553430000	55.9019650000
H106	10.6466870000	-0.3645340000	56.8186110000
C107	12.5942720000	-0.5937330000	54.8702930000
H108	13.1141270000	-1.1316070000	54.0687170000
H109	12.8813670000	-1.0542010000	55.8226680000
H110	12.9647920000	0.4414770000	54.8728410000
H111	13.4550770000	-12.2303890000	53.1883130000
C112	13.9110020000	-11.6210550000	52.4219880000
H113	14.6428840000	-10.9484170000	52.8820120000
H114	14.4000830000	-12.2515130000	51.6738150000
N115	12.8080270000	-10.8787180000	51.8067800000
H116	11.9685940000	-10.6943180000	52.3765590000
C117	12.8439160000	-10.2434400000	50.6424650000
N118	14.0212380000	-10.1449390000	49.9505830000
H119	13.9176670000	-9.6196690000	49.0882670000
H120	14.7627250000	-9.7292650000	50.5203210000
N121	11.7113810000	-9.7638320000	50.0944660000
H122	10.8846480000	-9.8086090000	50.7100290000
H123	11.8022070000	-8.8510430000	49.6368240000
H124	19.2228840000	-8.2219240000	61.6983190000
C125	19.5978940000	-7.2298800000	61.9900500000
H126	20.6379170000	-7.3507340000	62.3095220000
C127	18.8121540000	-6.7198090000	63.1928060000
O128	19.3495580000	-6.3459810000	64.2323490000
C129	19.4918720000	-6.2717980000	60.7990630000
H130	19.8766560000	-5.2857550000	61.0817880000
H131	18.4370790000	-6.1100430000	60.5527530000
C132	20.2089560000	-6.7719470000	59.5312700000
H133	20.0424770000	-7.8453180000	59.3850050000
H134	21.2969180000	-6.6443600000	59.6314340000
C135	19.7880010000	-6.1037210000	58.2184730000
O136	19.8411110000	-6.6923450000	57.1486210000
O137	19.3888530000	-4.8456170000	58.3766640000
N138	17.4645960000	-6.7025050000	62.9870730000
H139	17.1110500000	-6.9587360000	62.0738980000

C140	16.5368850000	-6.0058840000	63.8530420000
H141	15.8742430000	-6.7289710000	64.3511000000
H142	17.1343750000	-5.5168840000	64.6267600000
C143	15.6816870000	-4.9751850000	63.0808050000
H144	16.3283680000	-4.1874370000	62.6784560000
H145	15.0013420000	-4.5032600000	63.8024000000
C146	14.8903040000	-5.6106710000	61.9542220000
C147	13.7384670000	-6.3681850000	62.2172320000
H148	13.3753620000	-6.4505220000	63.2407040000
C149	15.3293840000	-5.5013630000	60.6254620000
H150	16.1891400000	-4.8729910000	60.4024970000
C151	13.0447390000	-7.0010310000	61.1853460000
H152	12.1436430000	-7.5682510000	61.4052100000
C153	14.6452310000	-6.1504240000	59.5919270000
H154	15.0026870000	-6.0712540000	58.5691740000
C155	13.4999620000	-6.9004760000	59.8672320000
H156	12.9602990000	-7.3879950000	59.0600670000
H157	9.0037560000	0.7050730000	60.7752690000
C158	8.1269790000	0.1560810000	60.4650000000
H159	7.2836720000	0.4592430000	61.0677700000
H160	14.8262490000	-4.8169690000	56.3255320000
H161	12.8564710000	-9.3844170000	54.8462880000
H162	18.2592780000	-9.4987530000	50.1505400000
C163	17.5839760000	-8.6559200000	50.1520250000
H164	17.2773630000	-8.4419890000	49.1387990000
H165	23.2192040000	-4.6257350000	52.5137160000
H166	10.6544200000	-6.1920750000	50.1665550000
O167	14.4668470000	-3.8481380000	56.5163630000
H168	14.7245160000	-3.3862510000	55.6861540000
H169	13.3654010000	-3.9858900000	56.3505210000

The TS structure with two waters and a proton on S1B.

Mo1	15.9660518745	-6.2395479202	53.2201957102
Fe2	8.0213216326	-8.1880553766	53.8545891299
Fe3	10.1506045637	-6.1787363313	54.5911407930
Fe4	10.5358662138	-7.5480279817	52.3071227136
Fe5	10.9321680547	-8.7180737224	54.5659925734
Fe6	13.5404815649	-8.1426782396	54.0920629856
Fe7	12.9137761528	-5.7478696390	54.8280454502
Fe8	13.0461096310	-6.3045269237	52.0834863552
C9	17.5565207959	-2.6173675826	54.2357473618
H10	17.8481498550	-1.6701008072	53.7806165750
H11	16.4593875212	-2.6456961287	54.2797581605
C12	18.0301523888	-3.7984847620	53.3587460437
C13	19.5673960701	-3.7394764311	53.1250468535
H14	20.0740508859	-3.8674524057	54.0884818679
H15	19.8127258167	-2.7400711876	52.7525152269

C16	20.0277437834	-4.8099503949	52.1303515226
H17	19.6316418741	-4.5948016576	51.1307966899
H18	19.5971103718	-5.7713041400	52.4131579576
C19	17.3309325471	-3.8439084951	51.9682722482
O20	16.7973447041	-4.9891197494	51.6570546033
O21	17.4121246450	-2.8563899587	51.2394576943
O22	17.6821512919	-5.0243473748	54.0394209565
C23	11.8908169764	-7.0989329315	53.6847358599
S24	9.2681072761	-7.9627989766	55.9679036984
S25	15.1500861251	-6.9991213821	55.5177611281
S26	8.6739864074	-6.0144537258	52.7134690674
S27	11.2658544270	-4.3834966395	55.6640999441
S29	14.2235125221	-4.4956798320	53.2222146839
S30	9.7462310975	-9.7486038706	52.6933383586
S31	14.7498151455	-8.0061981801	52.0341169846
S32	11.6247394449	-6.5949508065	50.1069899040
H33	13.6351747536#	-2.1902104907#	49.0425561624#
C34	13.2979254850#	-2.0241164721#	50.0550217977#
H35	13.9500856261	-2.5864243021	50.7290697805
H36	13.3234332924	-0.9593106891	50.3033317714
N37	11.9050381972	-2.5380200795	50.0666608880
H38	11.5635387655	-2.8588836586	49.1724356690
C39	11.1862470222	-2.9903124546	51.1243864014
N40	11.6960665066	-2.9975465419	52.3471989809
H41	11.1567851845	-3.3680094873	53.1348509228
H42	12.7044072261	-3.1167832534	52.4789692784
N43	9.9042943430	-3.3648336187	50.9455630945
H44	9.4943315157	-3.3066179671	50.0244345550
H45	9.4787154406	-4.0695293465	51.5811625457
H46	7.9230002793	0.3719439134	59.4109125858
C47	8.2362914640	-1.3611515670	60.7386169571
H48	8.4221085713	-1.5001908464	61.8112182843
H49	7.2480626559	-1.7992866849	60.5402822676
C50	9.2639033888	-2.1636867644	60.0019410527
N51	10.4940187259	-2.4540258460	60.5591245612
C52	9.1302307767	-2.8135343747	58.7931554207
H53	8.3294362882	-2.8550889295	58.0698047963
C54	11.0774621664	-3.2704825303	59.6976133651
H55	12.0402212860	-3.7471841085	59.8368656980
N56	10.2902073053	-3.5232521347	58.6171612538
H57	18.9230505982	-4.5449063512	57.5045690673
H58	16.7040714019	-8.8936287350	50.7457687512
C59	18.3772637109	-7.5214936578	50.8088829552
H60	17.8255923307	-6.5805522953	50.7725892241
H61	19.3259696897	-7.3684958875	50.2788861888
C62	18.6788215708	-7.8614569346	52.2405945827
N63	17.7518792923	-7.6563041968	53.2555734834
C64	19.8148438938	-8.3954746743	52.7993411089
H65	20.7665554328	-8.6548648088	52.3629821745
C66	18.3128071922	-8.0462578446	54.3871366162

H67	17.8621410950	-7.9748414418	55.3654000302
N68	19.5611797574	-8.5123449784	54.1562175450
H69	20.2193489212	-8.7475284956	54.8849068410
H70	6.3928523704#	-9.4883341743#	56.5258903355#
C71	6.1064156788	-10.0786495087	55.6487777746
H72	6.8824377624	-10.8282716479	55.4677993070
H73	5.1614613572	-10.5939092246	55.8555176255
S74	5.8710585871	-9.0041083833	54.1613478493
H75	10.5352026525	-4.0641987357	57.7776771247
H76	17.7702515622	-4.8231921164	55.0215678701
C78	18.0822471363	-2.6716144326	55.6826799389
O79	17.9688552201	-3.8457506254	56.2765595302
O80	18.5454635305	-1.6641757311	56.2214167338
C81	21.4973407649	-5.0859320799	51.9430032209
O82	21.9489444709	-5.9700762291	51.2367225166
O83	22.3208177968#	-4.2658020621#	52.6398598024#
H84	15.7024659160#	-0.1996312811#	61.4912310580#
C85	14.8549925897#	-0.0829919020#	60.8320024304#
H86	13.9165015895#	-0.2867338793#	61.3260937710#
H87	14.8295510878	0.9915883429	60.5848622136
C88	15.0599864919#	-0.8751942549#	59.5441988413#
H89	14.3905310545	-0.4956290541	58.7630924384
H90	14.7831657648	-1.9224869194	59.7162574683
C91	16.5009282938	-0.8559705150	59.0275338713
H92	16.9429984269	0.1451715630	59.1401170100
H93	16.5348213012	-1.0695881654	57.9526238215
C94	17.4052742267	-1.8660634997	59.7455676338
O95	17.0759176773	-2.4385711967	60.7860011033
N96	18.6170267486	-2.0570203677	59.1483185998
H97	19.1201658169	-2.8772271115	59.4666126224
H98	18.7093004214	-1.8494283390	58.1490233140
C99	10.6519927522#	0.0100037077#	53.3460011955#
H100	11.1636987212#	0.9592721955#	53.2872697307#
H101	9.5863320300#	0.1850132053#	53.3579376600#
H102	10.9214095069	-0.5867193681	52.4726621123
C103	11.0941131792	-0.6436990234	54.6681245928
H104	10.7795556047	-1.6928704990	54.6843722339
C105	10.4245362862	0.0470301621	55.8651725966
H106	9.3308909928	-0.0137898605	55.7974954232
H107	10.6990223721	1.1111650462	55.9146158327
H108	10.7220464687	-0.4220103008	56.8095126706
C109	12.6222616343	-0.6008046595	54.8018354574
H110	13.1139608940	-1.0845192596	53.9502505504
H111	12.9648321360	-1.1106958491	55.7067484985
H112	12.9752290544	0.4405763883	54.8422972336
H113	13.4550775183#	-12.2303887950#	53.1883134716#
C114	13.9110022627#	-11.6210552246#	52.4219874670#
H115	14.5858446299	-10.8887528885	52.8753178359
H116	14.4494937132	-12.2477143781	51.7054115594
N117	12.7733946855	-10.9695662348	51.7624710295

H118	11.9320488254	-10.7761268768	52.3296128275
C119	12.7711414553	-10.4127665849	50.5602090310
N120	13.9360944155	-10.3051199554	49.8553344936
H121	13.8365040444	-9.8133162859	48.9745096900
H122	14.6978919072	-9.9147691973	50.4125962455
N123	11.6034729433	-10.0405248307	49.9953912830
H124	10.7959305933	-10.0536483071	50.6431264538
H125	11.6282396310	-9.1672922606	49.4702821725
H126	19.1944023175	-8.2138675737	61.7117411260
C127	19.5978935120#	-7.2298798082#	61.9900498473#
H128	20.6401224067	-7.3702881937	62.2941140198
C129	18.8357783261	-6.6718105140	63.1911596627
O130	19.3898737349	-6.1512008919	64.1561936435
C131	19.4918718668#	-6.2717977658#	60.7990634447#
H132	19.9205511485	-5.2996359456	61.0654027175
H133	18.4361590625	-6.0795870841	60.5807863713
C134	20.1656567951	-6.8222539661	59.5263760064
H135	19.9563079809	-7.8919167046	59.4082105470
H136	21.2595358279	-6.7385958628	59.6028850438
C137	19.7488563138	-6.1748147520	58.2052728272
O138	19.8423296464	-6.7511156869	57.1328564065
O139	19.2917839745	-4.9322303506	58.3584856344
N140	17.4815945367	-6.7676418810	63.0552593763
H141	17.1137589327	-7.1515963967	62.1932949737
C142	16.5368852402#	-6.0058841663#	63.8530414299#
H143	15.9566175288	-6.6818113978	64.4977199344
H144	17.1209154941	-5.3497201225	64.5035486122
C145	15.5809311814	-5.1942807628	62.9522831609
H146	16.1484126286	-4.4237027019	62.4183149134
H147	14.8647771175	-4.6747089925	63.6030331532
C148	14.8368864158	-6.0683686917	61.9565907379
C149	13.8547076503	-6.9756514673	62.3891688316
H150	13.6041158460	-7.0275397647	63.4474631739
C151	15.1304864636	-6.0109777779	60.5848533301
H152	15.8634912184	-5.2927369181	60.2232711969
C153	13.1792801909	-7.7920323078	61.4812481445
H154	12.4086567675	-8.4725248931	61.8345913966
C155	14.4586758747	-6.8344111388	59.6722324380
H156	14.6951298166	-6.7763489217	58.6149160075
C157	13.4782235480	-7.7235617556	60.1169980067
H158	12.9441829914	-8.3450700484	59.4037613798
H159	9.0037565185#	0.7050726131#	60.7752692757#
C160	8.1269788035#	0.1560808373#	60.4650005935#
H161	7.2836719851#	0.4592428620#	61.0677695512#
H162	15.4038151498	-5.5952808627	56.5475393819
H164	12.6040058516	-9.5270653377	54.7308338005
H165	18.2592777766#	-9.4987531778#	50.1505404209#
C166	17.5839763734#	-8.6559200662#	50.1520247658#
H167	17.2773626136#	-8.4419892253#	49.1387987672#
H169	23.2245472382	-4.5690282098	52.4304817624

H170	10.6176331612	-5.7055353296	50.2151066404
O171	15.6593862465	-4.7547977888	57.2418832885
H172	14.9001405672	-4.0675748352	57.2319200437
H173	16.5053285352	-4.3239442399	56.9013598167
O174	13.7217317381	-3.0129168554	57.1950360969
H175	13.0012916622	-3.3974429048	56.6238620682
H176	13.2908245467	-2.8392651868	58.0483334339

The structure with a proton on S1A

Mo1	15.9338100271	-5.9747008965	53.2182212068
Fe2	8.0313234496	-7.6266346579	54.0054493296
Fe3	10.4489318658	-5.8066020821	54.7252675579
Fe4	10.5321159115	-7.1185210516	52.4404877008
Fe5	11.1569941599	-8.2840177812	54.6046841526
Fe6	13.7081235395	-7.7034105842	54.0549322763
Fe7	13.1818323508	-5.2329214648	55.0874669743
Fe8	13.0964780480	-5.8678849203	52.2956752311
C9	17.6795794703	-2.4512474235	54.2099331392
H10	18.0034549752	-1.5120182046	53.7579123884
H11	16.5851305520	-2.4211735386	54.2887503478
C12	18.0751317805	-3.6464813102	53.3190696440
C13	19.6131070373	-3.6667388912	53.0884565544
H14	20.0948765483	-3.8412433397	54.0554675414
H15	19.9166595827	-2.6740614707	52.7412310486
C16	20.0358201780	-4.7362677664	52.0750784661
H17	19.7305304834	-4.4417526392	51.0625278275
H18	19.5094780126	-5.6667690858	52.2838369487
C19	17.3818263397	-3.6783130422	51.9284523773
O20	16.8011194633	-4.8095400047	51.6327776541
O21	17.5056729461	-2.7176870130	51.1709861796
O22	17.6716030201	-4.8481066524	54.0123286878
C23	12.0380363387	-6.6755535193	53.8123522671
S24	9.3659694771	-7.9222001515	56.2246086281
S25	15.1938577804	-6.6587067798	55.5039861205
S26	8.7857627376	-5.5067221954	53.0299271241
S27	11.4013822705	-4.3142269900	56.1638824680
S29	14.3062346874	-4.0586949659	53.2706162551
S30	9.6614539720	-9.2659690753	52.9314706267
S31	14.6922653819	-7.6110807625	51.8970897857
S32	11.7021697115	-6.3320065408	50.2394657915
H33	13.6351748501#	-2.1902104232#	49.0425561834#
C34	13.2979254370#	-2.0241165157#	50.0550217044#
H35	13.8101332242	-2.7308991060	50.7124037429
H36	13.5202423764	-1.0004011503	50.3725545960
N37	11.8425039654	-2.2782223994	50.0476061389
H38	11.3450491479	-1.9732463915	49.2234351785
C39	11.0675763434	-2.8177789472	51.0071806283
N40	11.5086263134	-3.0903277835	52.2213947703

H41	10.9161572861	-3.6185227563	52.8621553521
H42	12.4982304794	-3.0470395635	52.4882769834
N43	9.7477769633	-3.0271141913	50.7353858730
H44	9.5063340647	-3.2043441269	49.7689407505
H45	9.2525391401	-3.6437122550	51.3942174435
H46	7.9320844740	0.3750229111	59.4100162680
C47	8.2990441092	-1.3572288436	60.7203359158
H48	8.2448996835	-1.5251834252	61.8043395599
H49	7.4450858277	-1.8894386602	60.2799587922
C50	9.5748502412	-1.9650997999	60.2310002579
N51	10.7311530051	-1.9145473250	60.9890027890
C52	9.8079102049	-2.6486921035	59.0603024930
H53	9.1816092575	-2.9095870960	58.2205322976
C54	11.6381261282	-2.5626808803	60.2790887323
H55	12.6649574841	-2.7366705302	60.5705255691
N56	11.1302210386	-3.0223703397	59.1056765245
H57	19.0055168959	-4.4810200220	57.4674379971
H58	16.6933434508	-8.9145668011	50.7282106227
C59	18.3151178542	-7.4896655157	50.8255686465
H60	17.7325080177	-6.5689307631	50.7441910253
H61	19.2769859700	-7.3139280169	50.3271519646
C62	18.5777671618	-7.7644326228	52.2790113813
N63	17.6400395947	-7.4903685697	53.2686991978
C64	19.7068066809	-8.2631809937	52.8834433213
H65	20.6669450872	-8.5347323944	52.4745004334
C66	18.1916637463	-7.8113819484	54.4277799339
H67	17.7398368031	-7.6666682423	55.3967560307
N68	19.4382366417	-8.2962671867	54.2414657810
H69	20.0903491604	-8.4440597364	54.9997396307
H70	6.3928523540#	-9.4883342477#	56.5258904137#
C71	6.1412671376	-9.7947459606	55.5037832359
H72	6.9465549863	-10.4264854144	55.1178782710
H73	5.2165238311	-10.3811726622	55.5262729481
S74	5.8691488065	-8.3280300329	54.4070244622
H75	11.5869809154	-3.5563953074	58.3662086415
H76	17.8366472317	-4.6340603312	55.0073278247
C78	18.2470143091	-2.5480980968	55.6494646945
O79	18.3146662199	-3.7444378836	56.1456281598
O80	18.5643207948	-1.5009911528	56.2367280277
C81	21.4895703482	-5.1168850041	51.9912616900
O82	21.9271536671	-6.0892759709	51.4004422628
O83	22.3208179827#	-4.2658020731#	52.6398598031#
H84	15.7024659475#	-0.1996312237#	61.4912310279#
C85	14.8549924681#	-0.0829919943#	60.8320024677#
H86	13.9165015895#	-0.2867338793#	61.3260937710#
H87	14.8245335529	0.9913624012	60.5820998896
C88	15.0599865637#	-0.8751942171#	59.5441988421#
H89	14.3532922616	-0.5306995762	58.7788200543
H90	14.8386392787	-1.9349843685	59.7164490240
C91	16.4914242853	-0.7708459722	58.9980550634

H92	16.8528187840	0.2667593139	59.0663394167
H93	16.5294927398	-1.0222586105	57.9319312602
C94	17.4903036011	-1.6734166470	59.7369812453
O95	17.2551955837	-2.1633302725	60.8471078967
N96	18.6675011348	-1.8692985514	59.0842738932
H97	19.2331158702	-2.6273407647	59.4477020066
H98	18.7207262535	-1.7120357557	58.0676028945
C99	10.6519924894#	0.0100039436#	53.3460013746#
H100	11.1636988319#	0.9592721273#	53.2872695942#
H101	9.5863320202#	0.1850131545#	53.3579375415#
H102	10.9125563056	-0.5669785141	52.4549754367
C103	11.0821152588	-0.6933394867	54.6468097645
H104	10.7472621603	-1.7372877456	54.6205654393
C105	10.4164121623	-0.0366689245	55.8646739411
H106	9.3221684545	-0.0624293807	55.7805161751
H107	10.7208923823	1.0156328403	55.9637901349
H108	10.6890589226	-0.5571593960	56.7887976895
C109	12.6079494688	-0.6936035338	54.8024112282
H110	13.1127679119	-1.1517591169	53.9443439545
H111	12.9081484160	-1.2613098273	55.6888601924
H112	12.9866854543	0.3340691068	54.9045869858
H113	13.4550772873#	-12.2303888849#	53.1883132627#
C114	13.9110026187#	-11.6210550894#	52.4219877437#
H115	14.6765849187	-10.9735872704	52.8623270015
H116	14.3352873586	-12.2449955846	51.6318205894
N117	12.7784740232	-10.8469939671	51.9007631185
H118	12.0200574550	-10.6246736602	52.5521661641
C119	12.7306088224	-10.2036613744	50.7403854988
N120	13.8513112803	-10.1256404433	49.9629907454
H121	13.7279739449	-9.5655866432	49.1262729960
H122	14.6774855735	-9.8207499798	50.4781629345
N123	11.5612511644	-9.7252248872	50.2881940004
H124	10.7766827465	-9.7488670971	50.9616243964
H125	11.5980426891	-8.8261382152	49.8044655929
H126	19.2027828531	-8.2161189103	61.7056930696
C127	19.5978935736#	-7.2298797969#	61.9900499046#
H128	20.6392298090	-7.3665856716	62.2989564151
C129	18.8273154557	-6.6985849091	63.1970808186
O130	19.3752358116	-6.2560390236	64.2036536266
C131	19.4918719302#	-6.2717978928#	60.7990634841#
H132	19.9248479313	-5.3001182805	61.0623213668
H133	18.4374113619	-6.0732964205	60.5809597236
C134	20.1524206834	-6.8160015123	59.5172308514
H135	19.9093564533	-7.8768218387	59.3805036993
H136	21.2482505511	-6.7719613625	59.6010123476
C137	19.7769958777	-6.1394110555	58.1917533036
O138	19.9190753491	-6.7154683002	57.1210794805
O139	19.3193882811	-4.9023881812	58.3392305839
N140	17.4727501763	-6.7498734402	63.0303704296
H141	17.1204829313	-7.0260859787	62.1220127145

C142	16.5368852045#	-6.0058841419#	63.8530416248#
H143	15.8214662124	-6.7015516342	64.3144159898
H144	17.1169561231	-5.5447150788	64.6562375517
C145	15.7728091409	-4.9384979744	63.0363651421
H146	16.4641436374	-4.1444034725	62.7340357581
H147	15.0263126219	-4.4810198068	63.7003304516
C148	15.0972684607	-5.5072004970	61.7998919438
C149	13.9910076277	-6.3669925050	61.8986428240
H150	13.5823152596	-6.6070854516	62.8792809277
C151	15.5932812635	-5.2001717890	60.5217497770
H152	16.4255404547	-4.5072696407	60.4305983760
C153	13.3929813652	-6.8989413375	60.7535966412
H154	12.5271281038	-7.5496596286	60.8482558732
C155	14.9976230736	-5.7347948391	59.3737308961
H156	15.3839222170	-5.4868437193	58.3892193123
C157	13.8952035499	-6.5857272825	59.4860727675
H158	13.4300736308	-6.9859247514	58.5900703214
H159	9.0037565753#	0.7050725797#	60.7752691737#
C160	8.1269785800#	0.1560807981#	60.4650009778#
H161	7.2836719626#	0.4592430746#	61.0677694131#
H162	9.5625884573	-6.7676443205	56.9063647716
H164	12.7624871821	-9.1489104051	54.5901555899
H165	18.2592776874#	-9.4987532494#	50.1505404424#
C166	17.5839763533#	-8.6559202097#	50.1520243042#
H167	17.2773626595#	-8.4419891642#	49.1387987661#
H169	23.2168234148	-4.6306283074	52.5111339339
H170	10.6449932270	-5.4938512161	50.1626881712

The TS structure with one water and a proton on S1A.

Mo1	15.9190910000	-6.0073400000	53.1683070000
Fe2	8.0858590000	-7.9027270000	53.7199760000
Fe3	10.3765670000	-6.0937480000	54.5034430000
Fe4	10.5747240000	-7.2373830000	52.1571810000
Fe5	11.1423080000	-8.5500490000	54.2392310000
Fe6	13.7069810000	-7.8330900000	53.8344530000
Fe7	13.1355150000	-5.3542210000	54.8931750000
Fe8	13.1115390000	-5.9370500000	52.1357410000
C9	17.6527990000	-2.4698800000	54.2220250000
H10	17.9920790000	-1.5387060000	53.7651090000
H11	16.5568690000	-2.4366130000	54.2741580000
C12	18.0621500000	-3.6773860000	53.3582390000
C13	19.6059130000	-3.7249540000	53.1814310000
H14	20.0461300000	-3.9738080000	54.1522740000
H15	19.9503030000	-2.7229090000	52.9080030000
C16	20.0332890000	-4.7389890000	52.1140120000
H17	19.7739260000	-4.3586780000	51.1167250000

H18	19.4759850000	-5.6643960000	52.2382030000
C19	17.4182470000	-3.7020340000	51.9471480000
O20	16.8341200000	-4.8244350000	51.6302420000
O21	17.5804780000	-2.7420830000	51.1965080000
O22	17.6135780000	-4.8669220000	54.0451040000
C23	12.0301630000	-6.8422760000	53.5941410000
S24	9.5129970000	-8.0593820000	55.8549600000
S25	15.0845990000	-6.7927450000	55.4094950000
S26	8.7755570000	-5.7072510000	52.7667650000
S27	11.3340610000	-4.5048220000	56.0357850000
S28	14.2742330000	-4.1045810000	53.1680200000
S29	9.7315310000	-9.4339470000	52.4688870000
S30	14.7654820000	-7.6128480000	51.7331080000
S31	11.8107920000	-6.3718080000	50.0216950000
H32	13.6351750000	-2.1902110000	49.0425560000
C33	13.2979250000	-2.0241160000	50.0550220000
H34	13.7994170000	-2.7350900000	50.7155700000
H35	13.5181610000	-1.0015360000	50.3765380000
N36	11.8375250000	-2.2723090000	50.0248790000
H37	11.3621870000	-1.9705360000	49.1866640000
C38	11.0441510000	-2.8760690000	50.9301210000
N39	11.4442840000	-3.1757780000	52.1537090000
H40	10.8512700000	-3.7726570000	52.7312950000
H41	12.4291910000	-3.1389560000	52.4401960000
N42	9.7422220000	-3.1120520000	50.6026870000
H43	9.5398970000	-3.2626540000	49.6229640000
H44	9.2394130000	-3.7615740000	51.2234370000
H45	7.9282450000	0.3907150000	59.4133610000
C46	8.3278750000	-1.3582500000	60.6820700000
H47	8.3239400000	-1.5548040000	61.7621480000
H48	7.4680760000	-1.8963660000	60.2611080000
C49	9.5992480000	-1.9166840000	60.1216100000
N50	10.7758390000	-1.8821970000	60.8483620000
C51	9.8156200000	-2.5175960000	58.9025290000
H52	9.1698050000	-2.7489130000	58.0687440000
C53	11.6788940000	-2.4575550000	60.0750310000
H54	12.7141040000	-2.6331090000	60.3312890000
N55	11.1500650000	-2.8526580000	58.8857840000
H56	18.9522550000	-4.4578070000	57.4985580000
H57	16.6971010000	-8.9114280000	50.7307840000
C58	18.3296250000	-7.4988690000	50.8272050000
H59	17.7637200000	-6.5685810000	50.7374910000
H60	19.2978190000	-7.3428170000	50.3347710000
C61	18.5793550000	-7.7736810000	52.2846880000
N62	17.6283630000	-7.5025860000	53.2629940000
C63	19.7016950000	-8.2681820000	52.9048480000
H64	20.6685890000	-8.5344950000	52.5087080000
C65	18.1665240000	-7.8161210000	54.4303260000
H66	17.7000110000	-7.6728470000	55.3925010000
N67	19.4173410000	-8.2956710000	54.2600220000

H68	20.0626030000	-8.4334240000	55.0259910000
H69	6.3928520000	-9.4883340000	56.5258900000
C70	6.2753130000	-9.9394040000	55.5333370000
H71	7.1783520000	-10.5116360000	55.3052570000
H72	5.4197390000	-10.6235090000	55.5518160000
S73	5.9623000000	-8.6522410000	54.2375210000
H74	11.6032280000	-3.3375390000	58.1126070000
H75	17.7393040000	-4.6498650000	55.0395480000
C76	18.1919570000	-2.5555620000	55.6696080000
O77	18.1968920000	-3.7386160000	56.2001280000
O78	18.5535860000	-1.5081190000	56.2302260000
C79	21.4785670000	-5.1486320000	52.0537910000
O80	21.9033170000	-6.1585900000	51.5206050000
O81	22.3208180000	-4.2658020000	52.6398600000
H82	15.7024660000	-0.1996310000	61.4912310000
C83	14.8549930000	-0.0829920000	60.8320020000
H84	13.9165020000	-0.2867340000	61.3260940000
H85	14.8184190000	0.9904950000	60.5779280000
C86	15.0599870000	-0.8751940000	59.5441990000
H87	14.3147490000	-0.5820540000	58.7941480000
H88	14.9143970000	-1.9459190000	59.7310140000
C89	16.4708850000	-0.6710310000	58.9624190000
H90	16.7125580000	0.4030670000	58.9464400000
H91	16.5222630000	-0.9999520000	57.9187680000
C92	17.5789030000	-1.3824960000	59.7553360000
O93	17.4712290000	-1.6828910000	60.9497350000
N94	18.7097980000	-1.6319350000	59.0477770000
H95	19.3836590000	-2.2339570000	59.5035920000
H96	18.7160180000	-1.6047480000	58.0176250000
C97	10.6519930000	0.0100040000	53.3460010000
H98	11.1636990000	0.9592720000	53.2872700000
H99	9.5863320000	0.1850130000	53.3579380000
H100	10.9129470000	-0.5732060000	52.4584370000
C101	11.0776900000	-0.6917490000	54.6493340000
H102	10.7137740000	-1.7270310000	54.6317490000
C103	10.4412860000	0.0010670000	55.8634570000
H104	9.3457910000	0.0032540000	55.7917360000
H105	10.7737260000	1.0466130000	55.9400340000
H106	10.7107950000	-0.5071320000	56.7952720000
C107	12.6048030000	-0.7317050000	54.7907410000
H108	13.0848230000	-1.2284530000	53.9402450000
H109	12.9003880000	-1.2827460000	55.6896330000
H110	13.0153400000	0.2860170000	54.8627940000
H111	13.4550770000	-12.2303890000	53.1883130000
C112	13.9110030000	-11.6210550000	52.4219880000
H113	14.5959210000	-10.9051600000	52.8898080000
H114	14.4475150000	-12.2542110000	51.7098130000
N115	12.8044720000	-10.9400870000	51.7448450000
H116	11.9775460000	-10.7018180000	52.3040430000
C117	12.8831850000	-10.3260630000	50.5683510000

N118	14.0760800000	-10.2878240000	49.9050670000
H119	14.0479780000	-9.7367200000	49.0544200000
H120	14.8520430000	-9.9873320000	50.4925830000
N121	11.7750740000	-9.8416480000	49.9812850000
H122	10.9311220000	-9.8160600000	50.5809210000
H123	11.8820890000	-8.9651150000	49.4703540000
H124	19.2123100000	-8.2179390000	61.6984690000
C125	19.5978940000	-7.2298800000	61.9900500000
H126	20.6372850000	-7.3615100000	62.3066050000
C127	18.8148580000	-6.7228160000	63.1977490000
O128	19.3505460000	-6.3502640000	64.2378220000
C129	19.4918720000	-6.2717980000	60.7990630000
H130	19.9343500000	-5.3037100000	61.0599160000
H131	18.4388440000	-6.0624250000	60.5887080000
C132	20.1327540000	-6.8114990000	59.5061120000
H133	19.8937840000	-7.8732400000	59.3709710000
H134	21.2296990000	-6.7588250000	59.5663650000
C135	19.7184130000	-6.1279710000	58.1949390000
O136	19.8077450000	-6.7072370000	57.1208010000
O137	19.2898250000	-4.8827220000	58.3590520000
N138	17.4614850000	-6.7265140000	63.0001980000
H139	17.1273290000	-6.9169080000	62.0639290000
C140	16.5368850000	-6.0058840000	63.8530420000
H141	15.7129720000	-6.6754080000	64.1366010000
H142	17.0862990000	-5.7476270000	64.7616120000
C143	15.9666950000	-4.7341150000	63.1809110000
H144	16.7642310000	-3.9883110000	63.0914140000
H145	15.2058610000	-4.3128120000	63.8520880000
C146	15.3732560000	-4.9935080000	61.8088440000
C147	14.1316880000	-5.6288700000	61.6517160000
H148	13.5562220000	-5.9111390000	62.5319660000
C149	16.0836310000	-4.6182700000	60.6583800000
H150	17.0171090000	-4.0754320000	60.7658580000
C151	13.6092900000	-5.8725280000	60.3790100000
H152	12.6308760000	-6.3335320000	60.2725380000
C153	15.5741410000	-4.8767210000	59.3824800000
H154	16.1401680000	-4.5811440000	58.5031560000
C155	14.3337780000	-5.5022120000	59.2409140000
H156	13.9419200000	-5.6804540000	58.2445670000
H157	9.0037570000	0.7050730000	60.7752690000
C158	8.1269790000	0.1560810000	60.4650010000
H159	7.2836720000	0.4592430000	61.0677690000
H160	11.3407040000	-5.9554140000	57.2697550000
H161	12.7903830000	-9.3190020000	54.3192680000
H162	18.2592780000	-9.4987530000	50.1505400000
C163	17.5839760000	-8.6559200000	50.1520240000
H164	17.2773630000	-8.4419890000	49.1387990000
H165	23.2127830000	-4.6447720000	52.5258080000
H166	10.7212470000	-5.5753440000	49.9401480000
O167	11.2626680000	-6.8901170000	57.7878310000

H168	10.4666700000	-7.3863360000	57.1025790000
H169	12.0868950000	-7.3638720000	57.5550390000
